# A thermophilic cell-free cascade enzymatic reaction for acetoin synthesis from pyruvate

**DOI:** 10.1038/s41598-017-04684-8

**Published:** 2017-06-28

**Authors:** Xiaojing Jia, Ying Liu, Yejun Han

**Affiliations:** 1 National Key Laboratory of Biochemical Engineering, Institute of Process Engineering, Chinese Academy of Sciences, Beijing, 100190 PR China; 2 University of Chinese Academy of Sciences, Beijing, 100049 PR China

## Abstract

Acetoin (3-hydroxy-2-butanone) is an important bio-based platform chemical with wide applications. *In vitro* enzyme catalysed synthesis exhibits great feasibility in the production of chemicals with high purity. In the present work, a synthetic pathway involving a two-step continuous reaction was constructed *in vitro* for acetoin production from pyruvate at improved temperature. Thermostable candidates, acetolactate synthase (coAHASL1 and coAHASL2 from *Caldicellulosiruptor owensensis* OL) and α-acetolactate decarboxylase (bsALDC from *Bacillus subtilis* IPE5-4) were cloned, heterologously expressed, and characterized. All the enzymes showed maximum activities at 65–70 °C and pH of 6.5. Enzyme kinetics analysis showed that coAHASL1 had a higher activity but lower affinity against pyruvate than that of coAHASL2. In addition, the activities of coAHASL1 and bsALDC were promoted by Mn^2+^ and NADPH. The cascade enzymatic reaction was optimized by using coAHASL1 and bsALDC based on their kinetic properties. Under optimal conditions, a maximum concentration of 3.36 ± 0.26 mM acetoin was produced from 10 mM pyruvate after reaction for 24 h at 65 °C. The productivity of acetoin was 0.14 mM h^−1^, and the yield was 67.80% compared with the theoretical value. The results confirmed the feasibility of synthesis of acetoin from pyruvate with a cell-free enzyme catalysed system at improved temperature.

## Introduction

Acetoin (3-hydroxy-2-butanone), with a pleasant creamy yogurt odour and fatty butter taste, abundantly exists in dairy foods and some fruits. It is widely used as a flavour and fragrance in the food industry^1^ and applied as an important intermediate in chemical synthesis and multifunctional materials^2, 3^. Due to its broad applications and great potential in large-scale industrial production, acetoin was classified as a promising bio-based platform chemical, and thus, its development and utilization was given priority by the U.S. Department of Energy^4^. As a result, methods for acetoin production have attracted extensive attention with its increasing demand in recent years. Currently, commercially available acetoin is largely produced by using chemical synthesis from fossil feedstocks, such as 2,3-butanediol (2,3-BD), butanone and diacetyl^1^. However, large-scale acetoin production has been limited due to the environmental problems from the chemical processes. An alternate clean method based on microbial fermentation and enzyme catalysis was biotechnological production^2^. However, the separation of acetoin was difficult in the microbial fermentative process because the end product is generally a mixture containing by-products such as 2,3-BD, acetic acid and lactic acid^2^. The separation of acetoin with high purity from the mixture is usually costly. Therefore, it is essential to construct an effective process to produce high-purity acetoin.

Many native microorganisms have been found to excrete acetoin as a physiological metabolite^5^. In bacterial metabolism, acetoin is formed from pyruvate through two enzyme-catalysed steps. The two enzymes involved are acetolactate synthase (ALS; EC 2.2.1.6) and α-acetolactate decarboxylase (ALDC; EC 4.1.1.5). ALS is a thiamine diphosphate (ThDP)-dependent enzyme that catalyses two pyruvate molecules to α-acetolactate or pyruvate and 2-ketobutyrate to acetohydroxybutyrate^6^. There are two types of ALS: anabolic acetohydroxyacid synthase (AHAS) and catabolic ALS (cALS)^7^. AHAS, commonly found in plants, fungi and bacteria, is applied in the biosynthesis of branched-chain amino acids (BCAAs) such as L-isoleucine, L-leucine and L-valine^6^. Meanwhile, cALS takes part in butanediol metabolism and is only found in some bacteria. Although they catalyse the same reaction, the properties of the two enzymes are different. Different from cALS, AHAS is flavin adenine dinucleotide (FAD)-dependent and contains a large catalytic subunit and a small regulatory subunit. In addition, AHAS is not specific for acetolactate production and yields 2-aceto-2-hydroxybutyrate from 2-ketobutyrate^7^. In the past decades, ALS has been purified and characterized from bacteria, including thermostable forms found in *Thermotoga maritima* ^8^ and *Bacillus stearothermophilus* ^9^.

Thereafter, α-acetolactate is decarboxylated by ALDC to acetoin, and CO_2_. ALDC has been applied in the traditional beer brewing process since, it could significantly increase the production rate and decrease the adverse flavour caused by diacetyl^10^. Apart from the catabolic degradation of α-acetolactate to acetoin in the 2,3-BD pathway, ALDC is also involved in the biosynthesis of BCAAs^11–13^. However, in some cases, the decarboxylation of α-acetolactate by ALDC is assumed to be a rate-limiting step in 2,3-BD biosynthesis^14, 15^. Mesophilic ALDC from *Bacillus brevis* ^16^, *Brevibacillus brevis* ^10^ and *Lactococcus lactis* subsp. *lactis* ^13, 17^ has been functionally characterized and structurally solved. Notably, no thermophilic ALDC has been reported yet.

Recently, cell-free biosystems using an enzyme cocktail have emerged as a powerful platform to synthesize compounds with high purity that cannot be obtained from chemical or fermentation processes^18^. Pyruvate, the key intermediate metabolite in the central carbon metabolic network, is classified as a highly attractive C3-buliding block for the synthesis of valuable chemicals^19^. In bacterial metabolism, a monosaccharide (hexose or pentose) is first converted to pyruvate, which is then converted to other attractive chemicals. The cell-free synthesis of acetoin has been reported by using glycerol and *meso*-2,3-BD as a substrate^20, 21^. Gao *et al*. also developed a NADPH-dependent system coupling carbonyl reductase and glucose dehydrogenase for acetoin production from diacetyl^22^.

**Figure 1:**
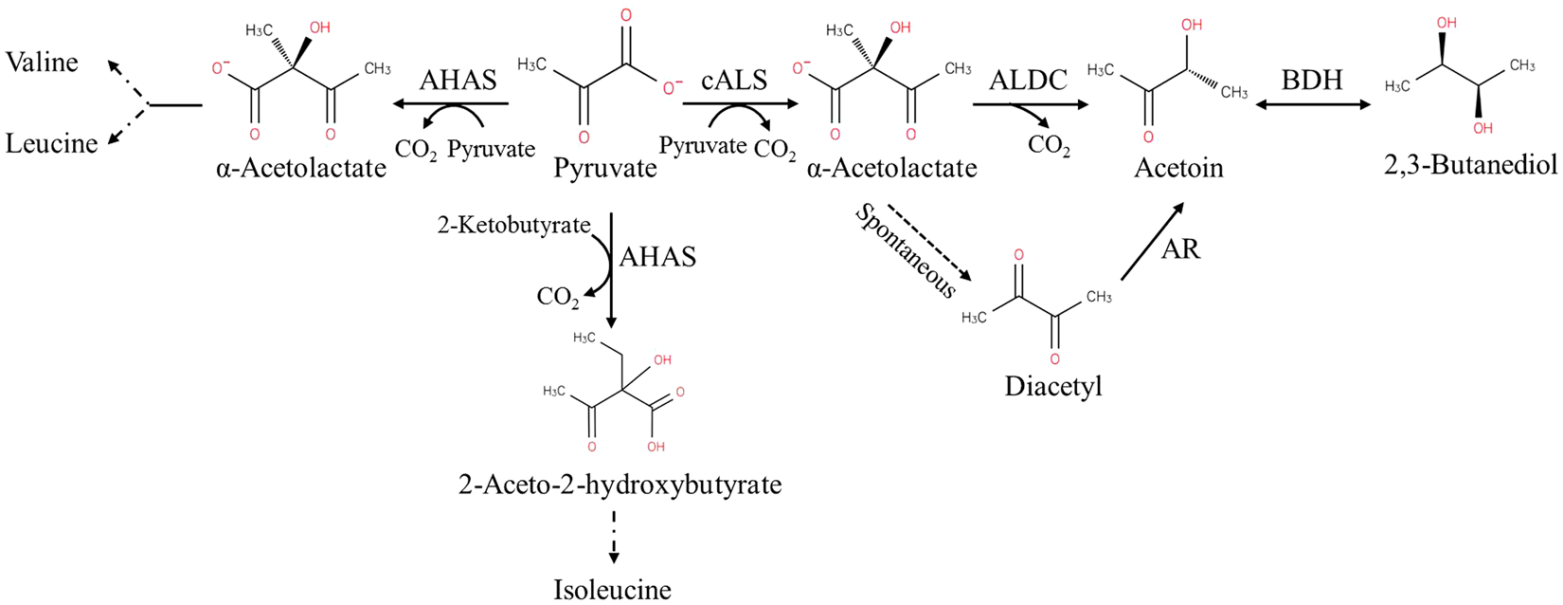
Scheme of the anabolic and catabolic pathways starting with pyruvate. In the figure: AHAS, anabolic acetohydroxyacid synthase; cALS, catabolic α-acetolactate synthase; ALDC, α-acetolactate decarboxylase; BDH, 2,3-butanediol dehydrogenase; and AR, diacetyl reductase.

In the present study, an *in vitro* artificial pathway involving two enzymes for acetoin production from pyruvate at high temperature was constructed (Fig. 1). Here is the chemical equation of the overall reaction occurring in the synthetic pathway:

$$2\,{{\rm{CH}}}_{3}{\rm{COCOOH}}+2\,{{\rm{H}}}_{2}{\rm{O}}\to {{\rm{CH}}}_{3}{\rm{COCH}}({\rm{OH}}){{\rm{CH}}}_{3}+2\,{{\rm{CO}}}_{2}$$

Thermophilic candidates for ALS and ALDC were cloned, heterologously expressed, and characterized. Then, the two thermostable enzymes were applied for acetoin production from pyruvate in a cell-free biosystem.

## Results

### Characterization of the enzymes for *in vitro* acetoin synthesis from pyruvate

#### ALS

Through analysis of the *Caldicellulosiruptor owensensis* genome, three genes were annotated as putative ALS enzymes. The full-length of the *coilvB* gene consists of an open reading frame (ORF) of 1611 bp and encodes a 58.63 kDa 536 amino acid protein. Compared with *coilvB*, the *coilvN* gene is much shorter in length, with an ORF of 513 bp encoding a 170 amino acid protein with a calculated molecular weight of 19.02 kDa. Similar to the *coilvB* gene, the *coilvI* gene is 1659 bp and encodes a 60.11 kDa protein consisting of 552 amino acid residues. Additionally, an identity of 48.0% was found between coAHASL1 and coAHASL2, which was much lower when comparing them with coAHASS (10.9–11.8%). No signal peptides were found in any of the three enzymes, indicating that they were intracellular enzymes. Sequence alignment by the BLAST similarity search in the GenBank database revealed coAHASL1 had 90–98% identity with ALS from *Caldicellulosiruptor* sp. and *Thermoanaerobacter cellulolyticus* [GenBank: WP_045166256.1], followed by 71% identity with ALS from *Pseudothermotoga thermarum* [GenBank: WP_013931525.1]. coAHASS shared up to 96–98% identity with enzymes from *Caldicellulosiruptor* sp. and *T*. *cellulolyticus* [GenBank: WP_045166257.1], followed by 78% identity with ALS from *Clostridium clariflavum* [GenBank: WP_014257070.1]. The coAHASL2 also had a 95–98% sequence identity conserved with proteins from *Caldicellulosiruptor* sp. and *T*. *cellulolyticus* [GenBank: WP_045166337.1], which was lowered to 68% when compared with the enzymes from *Clostridium* sp. Bc-iso-3 [GenBank: WP_069195743.1] and *Pseudobacteroides cellulosolvens* [GenBank: WP_036937152.1].

A conserved domain search revealed that both coAHASL1 and coAHASL2 were catalytic subunits of AHAS and belong to the thiamine pyrophosphate (TPP)-dependent family with the presence of the conserved TPP-enzyme-pyrimidine (PYR) superfamily, TPP-enzyme-M superfamily, and TPP-enzyme superfamily domains. On the other hand, coAHASS was a regulatory subunit of AHAS and belonged to the ACT domain family containing the ACT superfamily and ALS_ss_C superfamily domains. Genomic location analysis revealed that all three of the AHASs might play important roles in the biosynthetic pathway of BCAAs (Fig. S1A). Both the coAHASL1 and coAHASS genes were located not only in a *ilvB*-*ilvN*-*ilvC* operon with a *ilvC* gene encoding a ketol-acid reductoisomerase (KARI), which catalysed the following reaction after AHAS, but also near *leuA* genes encoding 2-isopropylmalate synthase (IPMS) in the L-leucine biosynthetic pathway. Similarly, coAHASL2 was located in the gene *ilvD* encoding a dihydroxyaciddehydratase (DHAD) and a gene encoding a branched-chain amino acid aminotransferase acting on L-isoleucine and L-valine. Furthermore, phylogenetic tree analysis clustered coAHASL1 and coAHASL2 together within the AHAS group, while coAHASS is distinct from them (Fig. S1B). Deep divergence among the AHAS large and small subunits, as well as cALS, was also revealed.

#### ALDC

Although the gene *alsD* encoding ALDC had been identified in some bacteria, the analysis of the *C*. *owensensis* genome did not reveal the existence of any homologue to *alsD*. Alternatively, we previously found a *Bacillus subtilis* strain IPE5-4 that displayed superior acetoin productivity from both glucose and xylose at temperatures up to 52 °C. Since it had the closest relationship with the strain *B*. *subtilis* 168, the primers for cloning the *bsalsD* gene were designed based on the nucleic acid sequence of *B*. *subtilis* 168 ALDC (BSU36000, GenBank: NP_391481.1). The gene sequence of bsALDC from *B*. *subtilis* IPE5-4 was deposited in GenBank with the accession number KY231247. Sequence analysis of bsALDC exhibited 99.2% identity with *B*. *subtilis* 168 BSU36000, suggesting the same function of bsALDC in α-acetolactate decarboxylation. The *bsalsD* gene was sequenced to be 768 bp encoding a 255 amino acid residue protein with a predicted molecular weight of 28.80 kDa. BLAST analysis of the deduced amino acid sequence of bsALDC revealed that it shared 60–99% identity with ALDC from *Bacillus* sp., followed by 59% identity with protein from *Sporolactobacillus* sp. Consistently, phylogenetic tree analysis clustered bsALDC and BSU36000 together, and they were located with *Bacillus licheniformis* ATCC 14580 ALDC [GenBank: AAU25289.1] (Fig. S1C).

### Expression and purification of the enzymes for *in vitro* acetoin synthesis from pyruvate

**Figure 2:**
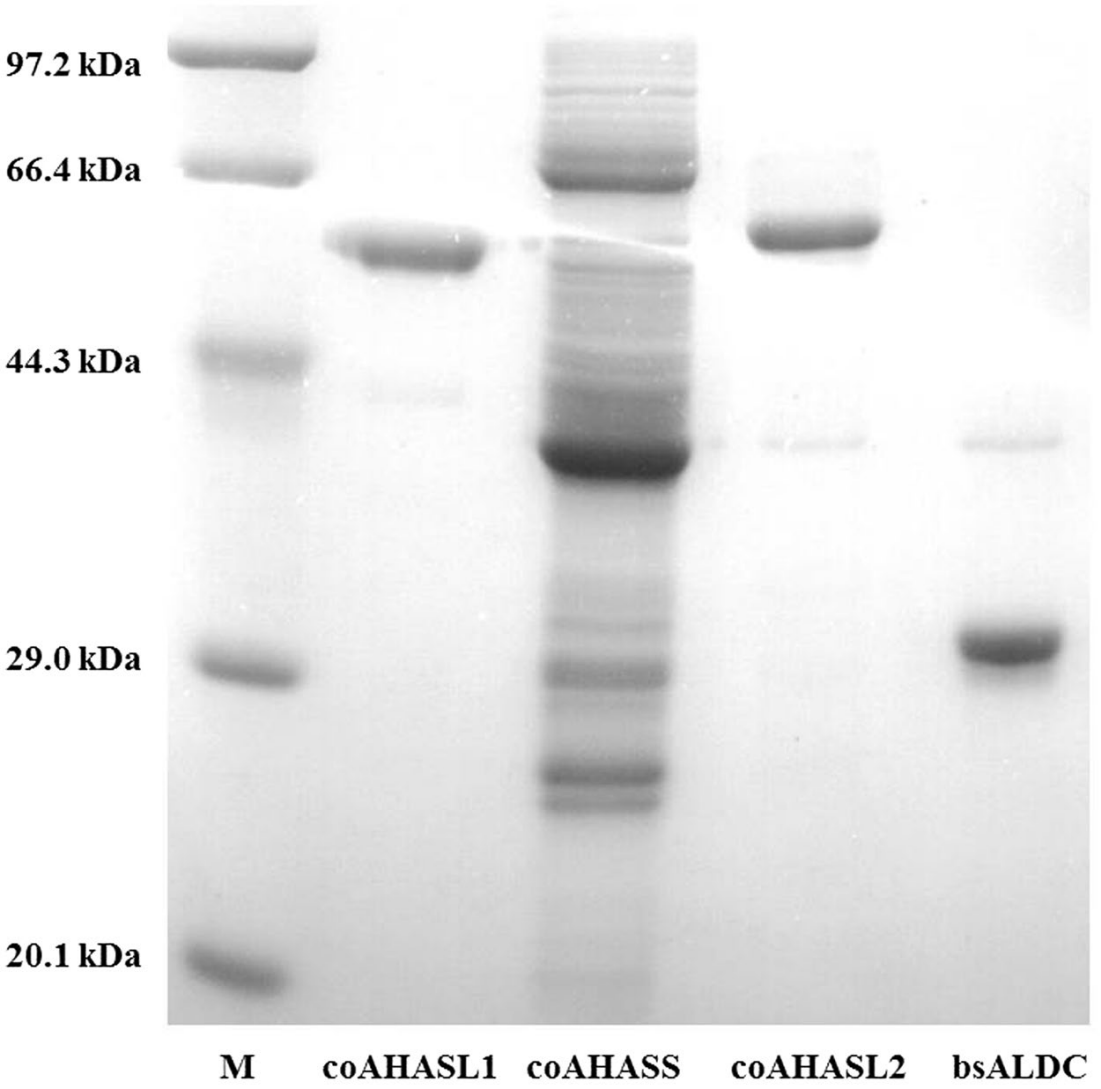
SDS-PAGE analysis of the recombinant enzymes involved in converting pyruvate to acetoin.

As shown in Fig. 2, the His-tagged recombinant ALS and ALDC were expressed in soluble forms after isopropyl-β-D-thiogalactopyranoside (IPTG) induction. The purified coAHASL1 and coAHASL2 proteins were observed as single bands on sodium dodecyl sulfphate-polyacrylamide gel electrophoresis (SDS-PAGE) with approximate molecular weights consistent with predicted values from the amino acid sequences (58.6 and 60.1 kDa, respectively). Similarly, the purified bsALDC protein was also observed as a single band on SDS-PAGE with a molecular weight of 28.8 kDa, which was consistent with *B*. *subtilis* 168 BSU36000. Meanwhile, coAHASS appeared as messy bands, and none of them corresponded to the molecular weight of 19.0 kDa.

### Selection of enzymes for *in vitro* acetoin synthesis from pyruvate

**Figure 3:**
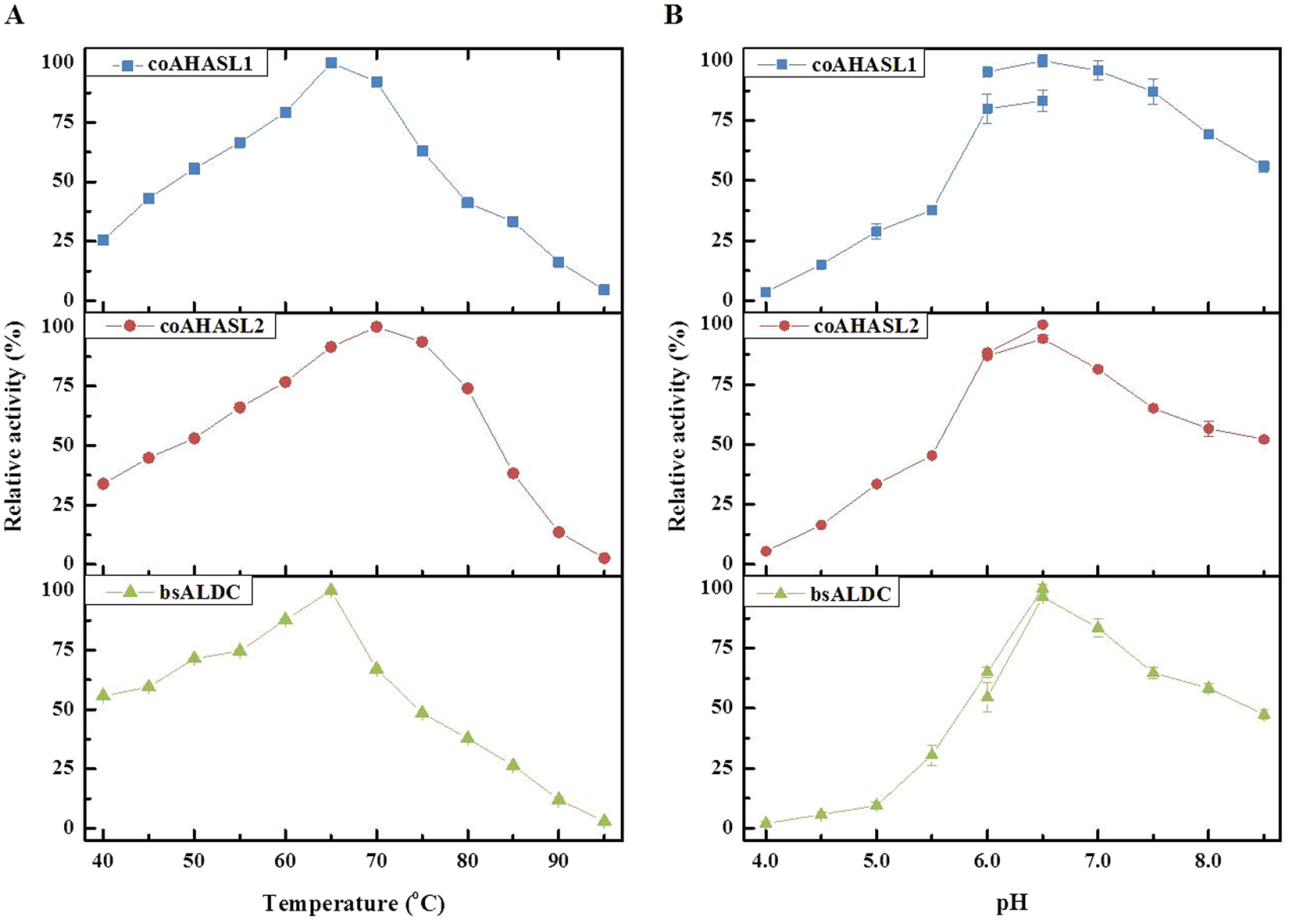
Temperature and pH profiles of the recombinant enzymes involved in converting pyruvate to acetoin. (**A**) Optimum temperatures of coAHASL1, coAHASL2 and bsALDC. (**B**) Optimum pH values of coAHASL1, coAHASL2 and bsALDC. Error bars indicate standard deviations of three independent experiments.

Activity screening exhibited that both coAHASL1 and coAHASL2 had catalytic properties accomplished with bsALDC, while no coAHASS activity was detected towards pyruvate. The temperature and pH profiles of each protein were characterized (Fig. 3). The optimum temperatures for coAHASL1, coAHASL2, and bsALDC were 65, 70, and 65 °C, respectively (Fig. 3A), and the optimum pH was determined to be 6.5 with Tris buffer for coAHASL1 and citrate-phosphate buffer for both coAHASL2 and bsALDC (Fig. 3B).

Subsequently, concentration-dependent enhancements of both coAHASL1 and coAHASL2 were observed upon the addition of FAD to the assay buffer, suggesting that the two catalytic subunits of ALS were FAD-dependent for activity. The results suggested that both coAHASL1 and coAHASL2 were AHAS on the basis of the structural and functional similarities with previously reported AHAS. In addition, the steady-state kinetic parameters of coAHASL1 and coAHASL2 were measured against pyruvate under the optimum conditions and compared with the previously reported catalytic subunit of AHAS (Table 1). Compared with coAHASL2, coAHASL1 displayed a lower affinity but higher activity against pyruvate than coAHASL2. Hence, coAHASL1 was the best candidate and chosen for the subsequent bioconversion *in vitro*. Meanwhile, the values of *V*_max_ and *K*_m_ with pyruvate by bsALDC were 2.34 ± 0.03 μmol min^−1^ mg^−1^ and 60.51 ± 2.67 mM, respectively, under the optimum conditions.

**Table 1:** Comparisons of the catalytic subunit of AHAS with pyruvate as a substrate.

Microorganism	Enzyme	Mw^a^ (kDa)	Optimum temperature (°C)	Optimum pH	*V*_max_^b^ (μmol min^−1^ mg^−1^)	*K*_m_^b^ (mM)	References
*C*. *owensensis*	coAHASL1	58.6	65	6.5	3.70 ± 0.06	71.83 ± 4.63	This study
*C*. *owensensis*	coAHASL2	60.1	70	6.5	0.70 ± 0.02	53.78 ± 2.68	This study
*T*. *maritima*	AHASL	66.4	85	7.0	246.00 ± 7.00	16.40 ± 2.00	8
*Thermus aquaticus*	AHAS	NA	70	8.0	NA^c^	NA	26
*Bacillus* sp.	AHAS	NA	75	6.0	NA	NA	26
*B*. *stearothermophilus*	AHASL	63.3	50	NA	3.00	12.00 ± 2.00	9
*E*. *coli*	AHAS I	90	37	7.0–7.5	20.80	0.95	33
*E*. *coli* K-12	AHAS I	65	37	7.5	1.50	4.15	34
*Pseudomonas aeruginosa* PA01	AHAS	65	37	7.5	0.12	14.20 ± 0.20	35
*Mycobacterium tuberculosis*	ilvB1 (Rv3003c)	68.3	35–40	7.0	2.80	2.76 ± 0.12	36
*Shigella sonnei*	AHAS	65	37	7.5	0.12	8.01	37

^a^MW, molecular weight by SDS-PAGE. ^b^The activity of each enzyme was determined under the optimal conditions with pyruvate as a substrate. ^c^NA, not available. ± indicates the standard deviation of three independent experiments.

Since the function of ALS requires a divalent metal ion apart from ThDP and FAD, effects of various metal ions and organic solvents on coAHASL1 and bsALDC activity were investigated in the presence of magnesium ions (Table 2). No activity was detected in the absence of Mg^2+^, and altering the concentration from 1 mM to 10 mM had 2-fold improvement on the activity of coAHASL1. Additionally, among all the ions tested, Mn^2+^ showed significant enhancement of both coAHASL1 and bsALDC activities, with 42.81% elevation for the coupled activity of ALS and ALDC when the concentration varied from 1 mM to 10 mM. In addition, K^+^, Ca^2+^, NH_4_^+^, and Ba^2+^ had little influence, and Co^2+^, Fe^2+^, and Cu^2+^ had negative effects on the activity. In contrast, Ni^2+^, Zn^2+^, Al^3+^, Cr^2+^, and Fe^3+^ displayed complete inhibition of enzyme catalysis. Moreover, NADH also greatly improved the activities of both coAHASL1 and bsALDC. Furthermore, NADPH, ATP, ADP, glycerol, DTT, NADP^+^, and NAD^+^ also had scant influence on the activity, while SDS and EDTA obviously annihilated the reaction. As a result, 10 mM Mn^2+^ was used as an additive in the following *in vitro* conversion based on the function test and economic feasibility.

**Table 2:** Effects of various metal ions and organic solvents on the activities of coAHASL1 and bsALDC.

Ion or reagent	coAHASL1	bsALDC
Control^a^	100.00 ± 1.19	100.00 ± 1.56
MnCl_2_	**168.79 ± 2.54**	**176.20 ± 2.34**
KCl	119.66 ± 0.34	88.23 ± 2.67
CaCl_2_	96.01 ± 1.98	123.68 ± 2.62
NH_4_Cl	110.29 ± 1.35	77.74 ± 1.04
BaCl_2_	108.39 ± 2.56	110.28 ± 1.61
CoCl_2_	66.97 ± 2.62	87.27 ± 2.54
FeCl_2_	54.86 ± 1.62	91.84 ± 2.74
NiCl_2_	61.58 ± 1.01	0.00 ± 0.63
CuCl_2_	52.78 ± 2.57	14.44 ± 1.43
ZnCl_2_	29.27 ± 0.42	0.00 ± 7.34
Al_2_(SO_4_)_3_	0.00 ± 2.34	0.00 ± 0.38
CrCl_2_	7.21 ± 0.51	13.29 ± 0.33
FeCl_3_	0.00 ± 0.76	0.15 ± 1.08
NADH	**199.72 ± 3.29**	**118.76 ± 0.68**
NADPH	121.71 ± 0.35	114.76 ± 0.93
ATP	112.10 ± 1.54	113.73 ± 3.04
Glycerol	90.34 ± 0.46	104.78 ± 0.19
DTT	77.07 ± 2.31	113.83 ± 4.55
NADP^+^	79.20 ± 2.82	95.01 ± 1.30
NAD^+^	68.00 ± 2.10	56.06 ± 0.66
ADP	67.37 ± 1.71	47.97 ± 3.69
SDS	0.00 ± 1.71	0.00 ± 0.17
EDTA	0.00 ± 1.56	0.13 ± 1.59

^a^100% was considered to be the activities of recombinant coAHASL1 and bsALDC without additives but in the presence of 1 mM MgCl_2_, respectively. ± indicates the standard deviation of three independent experiments.

### Optimization of the biocatalysis conditions

**Figure 4:**
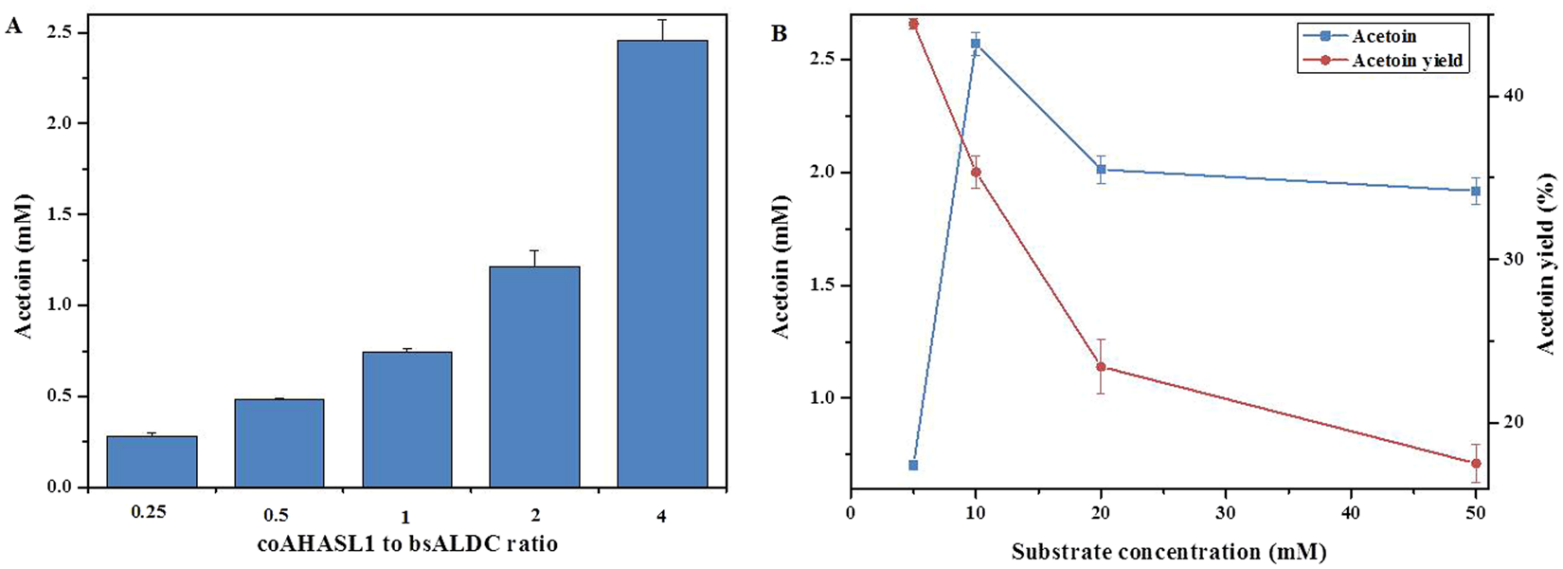
Optimization of the cell-free biosynthetic system. (**A**) Effect of the enzyme loading ratio on acetoin synthesis. Determination of the effect of the enzyme ratio on the synthesis of acetoin was tested in a total enzyme loading of 0.1 U mL^−1^ with 10 mM pyruvate. The ratio between coAHASL1 and bsALDC was changed from 0.25 to 4. (**B**) Effect of substrate loading on acetoin synthesis. Determination of the effect of the substrate concentration on the synthesis of acetoin was carried out by changing the concentration of sodium pyruvate from 5 to 50 mM. The total enzyme (coAHASL1/bsALDC 4:1) was applied at a ratio of 1 U/10 mM substrate. All reactions were conducted at 65 °C in citrate-phosphate buffer (pH = 6.5) for 12 h. Error bars indicate standard deviations of three independent experiments.

The effects of the enzyme loading ratio and pyruvate concentration on acetoin production were examined at 65 °C in citrate-phosphate buffer at a pH of 6.5 (Fig. 4). The total enzyme loading was 0.1 U mL^−1^, and the ratio between coAHASL1 and bsALDC was optimized from 0.25 to 4 (Fig. 4A). The conversion gradually increased from 17.38% to 36.73% as the coAHASL1/bsALDC ratio increased from 0.25 to 4. The highest production of acetoin was obtained at the coAHASL1/bsALDC ratio of 4:1. The effect of the substrate concentration on the synthesis of acetoin was also investigated by varying substrate concentrations from 5 to 50 mM at a fixed substrate/enzyme ratio (Fig. 4B). The acetoin titer increased from approximately 0.70 to 2.57 mM with increasing substrate concentrations from 5 to 10 mM. However, the product conversion decreased from 44.42% to 17.53% when the substrate concentration was increased from 5 to 50 mM. The higher substrate loading did not result in a higher production, possibly due to substrate or product inhibition.

### Synthesis of acetoin from pyruvate under the optimal conditions

**Figure 5:**
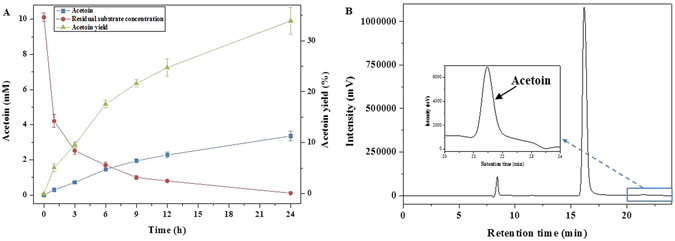
Production of acetoin from pyruvate under the optimal conditions. (**A**) Time course of the biocatalysis. (**B**) HPLC analysis of the products after reaction for 24 h. The reaction mixture contained 10 mM sodium pyruvate, 1 U total enzyme (coAHASL1/bsALDC 4:1), 0.5 mM ThDP, 10 μM FAD, 10 mM MgC1_2_ and 10 mM MnC1_2_. The reaction was conducted at 65 °C in 10 mL of citrate-phosphate buffer (pH = 6.5). Error bars indicate standard deviations of three independent experiments.

Combing the results, an optimal cell-free biosystem for acetoin production from pyruvate was constructed. The reaction was conducted at 65 °C in citrate-phosphate buffer at a pH of 6.5 containing 0.5 mM ThDP, 10 μM FAD, 10 mM MgC1_2_ and 10 mM MnC1_2_ at a total enzyme (coAHASL1/bsALDC 4:1) loading of 1 U/10 mM sodium pyruvate. As shown in Fig. 5, under the optimal conditions, 3.36 ± 0.26 mM acetoin was obtained from 10 mM pyruvate after 24 h of reaction, with a productivity and yield of 0.14 mM h^−1^ and 33.92%, respectively. Within 24 h, 98.94% of the substrate was converted to acetoin, reaching 67.80% of the theoretical value.

## Discussion

Thermostable enzymes are promising catalytic modules to construct more feasible bioconversion systems and for consolidated bioprocessing *in vitro* at high temperature. The biosynthesis of pyruvate to acetoin involves two enzymes: ALS and ALDC. ALS catalyses two molecules of pyruvate to generate one α-acetolactate, which is unstable and subsequently converted to acetoin by ALDC. However, the reports on thermophilic enzymes for acetoin biosynthesis are still limited. It has been confirmed that *Caldicellulosiruptor saccharolyticus* produced acetoin when grown in media supplemented with different monosaccharides, indicating that there were some candidate genes for acetoin formation in the genome^23^. While only putative *ilv* genes encoding ALS were identified in the genome of *Caldicellulosiruptor* sp. by bioinformatics analysis, the ALDC encoding gene *alsD* was not found^23^. As an alternative, an *alsD* gene was identified in the genome of *B*. *subtilis* IPE5-4, the catalytic role of which was confirmed in the conversion of pyruvate to acetoin.

Three putative ALS encoding genes were identified in the *C*. *owensensis* genome, and a homology search revealed that these three ALSs shared high identities with the other thermophile-derived ALSs. The comparison of coAHASL1 and coAHASL2 showed an even higher identity, indicating that they had the same function as the catalytic subunit of AHAS. In contrast, coAHASS shared limited identity with the catalytic subunit of coAHASL1 and coAHASL2, and it displayed no acetoin formation ability. Consistent with previous work, the genes encoding coAHASL1 and coAHASS are located within an *ilvB*-*ilvN*-*ilvC* operon^24^. The function of AHAS was also confirmed in the synthetic pathway for BCAAs by genetic analysis^7^. An activity assay revealed that the catalytic subunits coAHASL1 and coAHASL2 had their maximum activity at 65–70 °C at a pH of 6.5, which was consistent with other reported catalytic subunits^25, 26^. The two catalytic subunits showed both ThDP- and FAD-dependent catalytic activity, which was in line with the characteristics for the AHAS catalytic subunit^27^. Divalent ions such as Mg^2+^ and Mn^2+^ were essential for ALS activity. On the other hand, genes encoding cALS and ALDC are usually located with the 2,3-butanediol dehydrogenase (BDH)-encoding gene in an *alsSD* operon and regulated by AlsR in some gram-positive bacteria^5, 11^. Additionally, cALS is distinguished from anabolic AHAS due to its FAD-independent catalytic capacity without regulation by a small subunit^27^. It has been reported that acetoin accumulation is induced by conditions, such as a low external pH (5.5–6.5) and low oxygen levels, during the stationary phase^11^. The existence of cALS encoding genes in the genome of *Caldicellulosiruptor* sp. should be further studied, as should the presence of acetoin in the fermentation.

ALDC activity has been detected only among certain bacterial species, such as *Bacillus* sp. and *Lactobacillus* sp.^28^, and the ALDCs from different bacteria displayed different physical and chemical properties. In *B*. *subtilis*, the gene *alsS* encoding cALS and the gene *alsD* encoding ALDC belong to a single operon, which is controlled by the AlsR regulator^11^. The activation of ALDC by BCAAs has already been observed in microorganisms^12^. However, the ALDC from *L*. *lactis* subsp. *lactis* played dual roles in both the biosynthesis of BCAAs and acetolactate flux transfer to catabolism^13^. Redirecting the carbon flux of pyruvate in *Candida glabrata* to accumulate acetoin suggested that ALDC was rate-limiting for producing acetoin^14^. The overexpression of *Enterobacter aerogenes* ALDC in pyruvate decarboxylase-deficient *Saccharomyces cerevisiae* improved the 2,3-BD production^15^. The recombinant bsALDC was most active at 65 °C and a pH of 6.5, and it had a slightly higher affinity and lower activity towards pyruvate compared with coAHASL1. It was thus especially noteworthy that the thermophilic activity of bsALDC made it possible to produce acetoin *in vitro* at high temperature.

High-purity acetoin has been in huge demand as a precursor to synthesize novel chemicals and multifunctional materials^2, 3^. Hence, the ability to biologically produce acetoin with a high purity is crucial for improving its commercial viability. In acetoin fermentation from natural microorganisms, such as *Enterobacter* sp., *Bacillus* sp., and lactic acid bacteria, the mixture contains many by-products, and a large amount of them were fluxed to the end-product 2,3-BD by BDH^5^. The extraction process is environmentally unfriendly and costly. In contrast, enzymatic conversion is popular due to the higher chemical selectivity, more modest reaction conditions and lower levels of chemical contaminants^20^. Moreover, the *in vitro* approach increases the molecular efficiency by eliminating undesired metabolic side-reactions and does not require the complex preparation procedures of biocatalysts from growth medium. Pyruvate is an important central intermediate from which other biomolecules can be formed through a few additional catalytic steps. *In vitro* acetoin synthesis from pyruvate potentially offers a yield of 50.03% and allows simplified product recovery procedures. The previous report of cell-free acetoin production from glycerol was also based on pyruvate generation, in which 4.4 mM acetoin was generated from 10.4 mM glycerol with 85.5% of the theoretical yield at 50 °C after 24 h^20^. Through a thermophilic process consecutively catalysed by ALS and ALDC, 3.36 ± 0.26 mM acetoin was obtained from 10 mM pyruvate, with a productivity and yield of 0.14 mM h^−1^ and 33.92%, respectively. Compared with the work by Gao *et al*., the much higher activities of ALS and ALDC from *B*. *licheniformis* evidently contributed to the higher yield of acetoin^20^.

Advances in synthetic and systems biology have allowed for improvements in the efficacy of *in vitro* synthetic biosystems in terms of higher robust reaction rates and product yields. The entire processes can be improved with some biotechnological methods, i.e., enzyme engineering and process engineering^18^. The strategies of rational design, directed evolution, or a combination offer efficient and powerful tools to improve and optimize natural enzymes for generating robust biocatalysts for practical applications^29, 30^. Enzyme co-immobilization technologies insure the stability, recovery and reuse of the cell-free biosystem for several consecutive batches^31^. In addition, the process could be enhanced *via* optimization of the enzymatic components involved in the metabolic pathway by nonlinear kinetic modelling, metabolic flux analysis, and reaction control, i.e., substrate concentration, enzyme loading, and elevated temperature^32^. It is likely that the performance and reusability of the process studied in this work might be improved by enzyme engineering or co-immobilization in future works. Since glucose can be theoretically converted to pyruvate *via* the glycolysis pathway, the applicability of this approach addressed the viability of recruiting an *in vitro* highly interconnected pathway from central carbon metabolism for the purpose of synthesizing acetoin.

In a conclusion, a thermophilic cascade enzymatic reaction was constructed for *in vitro* acetoin production from pyruvate. Thermostable candidates of ALS and ALDC were cloned, purified and characterized. Subsequently, the bioconversion conditions, including the enzyme loading ratio and substrate concentration, were optimized with coAHASL1 and bsALDC at 65 °C. Herein, a maximum concentration of 3.36 ± 0.26 mM acetoin was obtained from 10 mM pyruvate after reaction for 24 h, with a productivity of 0.14 mM h^−1^ and yield of 33.92%. Based on the great engineering flexibility of cell-free bio-systems, this synthetic pathway originating from pyruvate offered enormous potential to serve as a versatile bio-system for chemical synthesis.

## Materials and Methods

### Chemicals

Sodium pyruvate, ThDP and FAD were purchased from Aladdin (USA). Acetoin and other chemicals, unless stated otherwise, were obtained from Sinopharm (Beijing, China). All other chemicals were of analytical grade.

### Bacterial strains, primers and plasmids

Bacterial strains, plasmids, and primers used in this study are listed in Table S1. *C*. *owensensis* OL was anaerobically cultured in DSMZ 640 medium at 70 °C, and used for cloning the genes *coilvB*, *coilvN* and *coilvI*. *B*. *subtilis* IPE5-4 was used for *bsalsD* cloning. Lysogeny broth (LB) medium was used for *Escherichia coli* and *B*. *subtilis* IPE5-4 culture. PCR primers were synthesized by Sangon (Shanghai, China).

### Gene cloning, protein expression and purification

Genomic DNA of *C*. *owensensis* OL and *B*. *subtilis* IPE5-4 were extracted by using a TIANamp Bacteria DNA Kit (TianGen, Beijing, China). Genes *coilvB* (Calow_0356, GenBank: ADQ03944.1), *coilvN* (Calow_0357, GenBank: ADQ03945.1), and *coilvI* (Calow_0543, GenBank: ADQ04125.1) were cloned from the genomic DNA of *C*. *owensensis*, and *bsalsD* was cloned from the genomic DNA of *B*. *subtilis* IPE5-4. PCR reactions were performed with Pfu DNA polymerase (TianGen) with the procedures listed below: 95 °C for 5 min, 30 cycles of 95 °C for 30 s, 57 °C for 1 min, 72 °C for 1 min, and finally 72 °C for 5 min. The PCR product was purified using a TIAN gel Midi Purification Kit (TianGen) and treated with T4 DNA polymerase (Takara, Dalian, China) at 37 °C for 30 min. After inactivation at 75 °C for 20 min, the treated genes were inserted into the pET-28b EK/LIC vector at 22 °C for 20 min, transformed into *E*. *coli* Top10 competent cells, and grown overnight on an LB agar plate with kanamycin (50 μg mL^−1^) at 37 °C. Positive clones were confirmed by colony PCR and DNA sequencing.

For each enzyme, recombinant plasmid was extracted by using a TIANprep Mini Plasmid Kit (TianGen) and transformed into *E*. *coli* BL21(DE3) competent cells for protein expression. A single colony was respectively picked and inoculated overnight at 37 °C in liquid medium with 50 μg mL^−1^ kanamycin. Protein production was induced in 1 L of liquid medium containing 50 μg mL^−1^ kanamycin by adding 0.5 mM IPTG at the logarithmic phase at 37 °C for 4–6 h at the OD600 of 0.4–0.6. Cells were harvested by centrifugation at 4000 rpm and 4 °C for 20 min. Precipitate was resuspended in 30 mL of binding buffer (50 mM Tris-HCl, 150 mM NaCl, pH = 6.0) and broken by ultrasonication. The cell lysate was centrifuged at 12000 rpm and 4 °C for 20 min, and the lysed supernatant was heat-treated at 50 °C for 30 min to remove thermally unstable proteins. The heat-treated lysate was then centrifuged at 12000 rpm and 4 °C for another 20 min, and the soluble extract was collected and loaded onto a pre-equilibrated His-tag Ni-affinity resin column (National Engineering Research Centre for Biotechnology, Beijing, China). The column was first washed with binding buffer to remove non-associative proteins, and then it was eluted with 5 mL of elution buffer (50 mM Tris-HCl, 150 mM NaCl, and 500 mM imidazole, pH = 6.0). The fractions were concentrated by ultrafiltration at 3000 rpm and 4 °C for 30 min. The purified protein was confirmed by SDS-PAGE. The protein concentration was also determined by the Bradford method using bovine serum albumin (BSA) as a standard. All the assays were performed in triplicate.

### Enzyme activity assay

For the activity assay of ALS, a standard mixture containing 20 mM sodium pyruvate, 0.5 mM ThDP, 10 μM FAD, and 10 mM MgC1_2_ in 100 μL of Tris buffer (50 mM Tris-HCl, 150 mM NaCl, pH = 6.0) was prepared. The assay mixture was pre-incubated at 60 °C for 5 min, and 5 μL of enzyme was added to start the reaction. The reaction mixture was maintained at 60 °C for 5 min and then quenched by the addition of 5 μL of 4 M H_2_SO_4_. The subsequent decarboxylation of acetolactate into acetoin was conducted by incubation at 60 °C for 15 min. The colour was developed with the addition of 90 μL of 0.5% (*w*/*v*) creatine and 90 μL of 5% (*w*/*v*) α-naphthol (in 2.5 M NaOH, freshly prepared) followed by incubation at 60 °C for 15 min. The resulting product was cooled for another 15 min at room temperature and measured at 530 nm. One specific unit of ALS activity was defined as the amount of enzyme required to produce 1 μmol of acetoin per minute. Each determination was run at least in triplicate.

The activity of ALDC was determined with coAHASL1 as described for ALS with the exception of acidification. The concentration of acetoin produced by ALDC was measured against a standard curve by using acetoin. One unit of ALDC activity corresponds to the formation of 1 μmol of acetoin per milligram of total protein per minute.

The optimum temperatures of ALS and ALDC were determined within a temperature range from 40 to 95 °C in 100 μL of Tris buffer (pH = 6.5). Subsequently, the optimum pH was measured in buffers with different pH values (citrate-phosphate 4.0–6.5, Tris 6.0–8.5) at 50 °C. Moreover, the effect of FAD activation on the ALS activity was analysed in the presence of 0–10 μM FAD at 65 °C in 100 μL of Tris buffer (pH = 6.5). In addition, the effects of metal ions and organic solvents on coAHASL1 and bsALDC activities were analysed with 1 mM Mg^2+^ at 65 °C in 100 μL of Tris buffer (pH = 6.5). The kinetic parameters *V*_max_ and *K*_m_ of ALS and ALDC were analysed by non-linear regression using GraphPad Prism version 6.0 for Windows (GraphPad Software Inc., USA).

### Conversion of pyruvate to acetoin *in vitro*

The enzymes coAHASL1 and bsALDC were used for the production of acetoin from pyruvate. Apart from sodium pyruvate, the reaction mixture also contained 0.5 mM ThDP, 10 μM FAD, 10 mM MgC1_2_ and 10 mM MnC1_2_ in 10 mL of citrate-phosphate buffer (pH = 6.5). The biocatalytic parameters including the reaction enzyme ratio and pyruvate loading for acetoin production were optimized. The reaction catalysed by coAHASL1 and bsALDC was carried out at a fixed enzyme loading of 0.1 U mL^−1^. The effect of the enzyme ratio was tested with coAHASL1/bsALDC ratios (U U^−1^) of 1:1, 1:2, 1:4, 2:1, and 4:1. The effect of the substrate concentration on the synthesis of acetoin was tested by varying the sodium pyruvate concentration from 5 to 50 mM. The total enzyme loading was kept at a ratio of 1 U/10 mM substrate with a coAHASL1/bsALDC ratio of 4:1. All the reactions were conducted at 65 °C for 12–24 h with at least three replicates. Samples were taken periodically and centrifuged at 12000 rpm for 15 min. The concentrations of residual pyruvate and produced acetoin were further analysed.

### Product analysis

The amounts of pyruvate and acetoin were quantified by a high-performance liquid chromatography instrument (HPLC, Shimadzu, Japan) equipped with a refractive index detector (LC-20AT, Shimadzu) and a Hi-PlexH exclusion column (300 × 7.7 mm, Agilent, United Kingdom). The column temperature was maintained at 65 °C with an injection volume of 10 μL. Milli-Q filtered 0.005 M H_2_SO_4_ was used as the mobile phase at a flow rate of 0.6 mL min^−1^.

## Electronic supplementary material

Supplementary Information
