# Supplementary material for: A thermophilic cell-free cascade enzymatic reaction for acetoin synthesis from pyruvate
Source: Sci Rep. 2017 Jun 28;7:4333. doi: 10.1038/s41598-017-04684-8 (PMC5489476; doi:10.1038/s41598-017-04684-8)
Supplement: Supplementary file 1 — Supplementary Information [file 41598_2017_4684_MOESM1_ESM.pdf]

A thermophilic cell-free cascade enzymatic reaction for acetoin synthesis from pyruvate

Xiaojing Jia[1,2], Ying Liu[1] & Yejun Han[1]

[1]National Key Laboratory of Biochemical Engineering, Institute of Process Engineering, Chinese Academy of Sciences, Beijing 100190, PR China. [2]University of Chinese Academy of Sciences, Beijing 100049, PR China. Correspondence and requests for materials should be addressed to Y. H. (email: yjhan@ipe.ac.cn)

Table

Strains, plasmids and primers	Properties and sequences	Sources
Strains		
Caldicellulosiruptor owensensis OL	DSM 13100, wild type	DSMZ
Bacillus subtilis IPE5-4	Wild type	Lab stock
Escherichia coli Top10	Cloning host	TianGen
E. coli BL21(DE3)	Expression host	Novagen
Plasmids		
pET28b	Overexpression vector; Kan^r	Novagen
pET28b-coAHASL1	pET28b with *coilvB* gene of *C. owensensis*	This study
pET28b-coAHASS	pET28b with *coilvN* gene of *C. owensensis*	This study
pET28b-coAHASL2	pET28b with *coilvI* gene of *C. owensensis*	This study
pET28b-bsALDC	pET28b with *bsalsD* gene of *B. subtilis* IPE5-4	This study
Primers (5'-3')		
coilvB-F	GACGACGACAAGatgaaggtgaagatgacggtagcac	This study
coilvB-R	GAGGAGAAGCCCGGttaaggtatagttggctcatcag	This study
coilvN-F	GACGACGACAAGatgaaatatacactttcagttttggttg	This study
coilvN-R	GAGGAGAAGCCCGGttactcatcctcctcagacttag	This study
coilvI-F	GACGACGACAAGatgaaattaacaggagctgaaattataatc	This study
coilvI-R	GAGGAGAAGCCCGGttaatcaataatctcatcaattggcg	This study
bsalsD-F	GACGACGACAAGatgaaacgmgaaagcaayattcaagtgct	This study
bsalsD-R	GAGGAGAAGCCCGGttattcmgggcttccttcrgttgtttc	This study

Table S1. Strains, plasmids, and primers used in this study.

Figure Legends

Figure S1. Genomic organization and phylogenetic tree analysis of ALS and ALDC. (**A**) Genomic organization of the various *ilv* genes encoding ALS in *C. owensensis*. (**B**) Phylogenetic tree of *C. owensensis* ALS. (**C**) Phylogenetic tree of *B. subtilis* IPE5-4 ALDC. Abbreviations: AHASL, catalytic subunit of acetohydroxyacid synthase; AHASS, regulatory subunit of acetohydroxyacid synthase; KARI, ketol-acid reductoisomerase (EC 1.1.1.86); IPMS, 2-isopropylmalate synthase (EC 2.3.3.13); DHAD, dihydroxyaciddehydratase (EC 4.2.1.9). The trees were constructed using MEGA 5.05 with 1000 bootstrap values, and GenBank accession numbers of each protein were listed after scientific names.

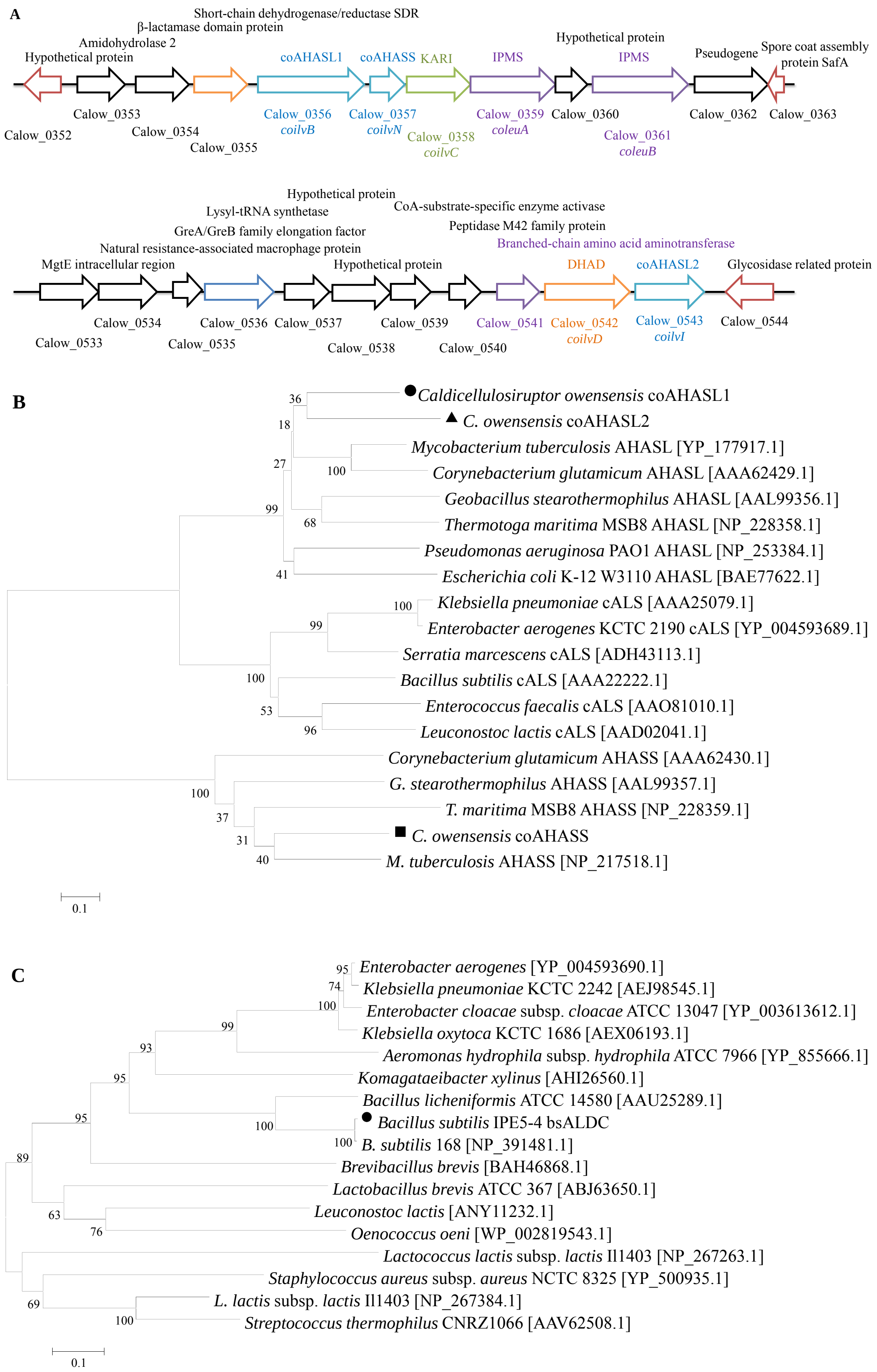
A
Short-chain dehydrogenase/reductase SDR
β-lactamase domain protein
Amidohydrolase 2
Hypothetical protein
coAHASL1
coAHASS
KARI
IPMS
Hypothetical protein
IPMS
Pseudogene
Spore coat assembly protein SafA
Calow_0352
Calow_0353
Calow_0354
Calow_0355
Calow_0356 coilvB
Calow_0357 coilvN
Calow_0358 coilvC
Calow_0359 coleuA
Calow_0360
Calow_0361 coleuB
Calow_0362
Calow_0363
Hypothetical protein
Lysyl-tRNA synthetase
CoA-substrate-specific enzyme activase
GreA/GreB family elongation factor
Peptidase M42 family protein
Natural resistance-associated macrophage protein
Branched-chain amino acid aminotransferase
MgtE intracellular region
Hypothetical protein
DHAD
coAHASL2
Glycosidase related protein
Calow_0533
Calow_0534
Calow_0535
Calow_0536
Calow_0537
Calow_0538
Calow_0539
Calow_0540
Calow_0541
Calow_0542 coilvD
Calow_0543 coilvI
Calow_0544
B
Caldicellulosiruptor owensensis coAHASL1
C. owensensis coAHASL2
Mycobacterium tuberculosis AHASL [YP_177917.1]
Corynebacterium glutamicum AHASL [AAA62429.1]
Geobacillus stearothermophilus AHASL [AAL99356.1]
Thermotoga maritima MSB8 AHASL [NP_228358.1]
Pseudomonas aeruginosa PAO1 AHASL [NP_253384.1]
Escherichia coli K-12 W3110 AHASL [BAE77622.1]
Klebsiella pneumoniae cALS [AAA25079.1]
Enterobacter aerogenes KCTC 2190 cALS [YP_004593689.1]
Serratia marcescens cALS [ADH43113.1]
Bacillus subtilis cALS [AAA22222.1]
Enterococcus faecalis cALS [AAO81010.1]
Leuconostoc lactis cALS [AAD02041.1]
Corynebacterium glutamicum AHASS [AAA62430.1]
G. stearothermophilus AHASS [AAL99357.1]
T. maritima MSB8 AHASS [NP_228359.1]
C. owensensis coAHASS
M. tuberculosis AHASS [NP_217518.1]
36
18
27
100
99
68
41
100
99
100
53
96
100
37
31
40
0.1
C
Enterobacter aerogenes [YP_004593690.1]
Klebsiella pneumoniae KCTC 2242 [AEJ98545.1]
Enterobacter cloacae subsp. cloacae ATCC 13047 [YP_003613612.1]
Klebsiella oxytoca KCTC 1686 [AEX06193.1]
Aeromonas hydrophila subsp. hydrophila ATCC 7966 [YP_855666.1]
Komagataeibacter xylinus [AHI26560.1]
Bacillus licheniformis ATCC 14580 [AAU25289.1]
Bacillus subtilis IPE5-4 bsALDC
B. subtilis 168 [NP_391481.1]
Brevibacillus brevis [BAH46868.1]
Lactobacillus brevis ATCC 367 [ABJ63650.1]
Leuconostoc lactis [ANY11232.1]
Oenococcus oeni [WP_002819543.1]
Lactococcus lactis subsp. lactis Il1403 [NP_267263.1]
Staphylococcus aureus subsp. aureus NCTC 8325 [YP_500935.1]
L. lactis subsp. lactis Il1403 [NP_267384.1]
Streptococcus thermophilus CNRZ1066 [AAV62508.1]
95
74
100
99
93
95
95
100
100
89
63
76
69
100
0.1

Figure S1
